# Post-traumatic tremor in child after mild head trauma

**DOI:** 10.11604/pamj.2018.31.53.16991

**Published:** 2018-09-25

**Authors:** Ploutarchos Karydakis, Dimitrios Giakoumettis, Ioannis Nikas, Dimitrios Panagopoulos, George Sfakianos, Marios Themistocleous

**Affiliations:** 1 Department of Neurosurgery, 251 Hellenic Air Force General Hospital, Athens, Greece; 2 Department of Neurosurgery, University of Athens, Evangelismos Hospital, Athens, Greece; 3 Department of Medical Imaging and Interventional Radiology, Agia Sofia Children’s Hospital, Athens, Greece; 4 Department of Neurosurgery, Agia Sofia Children’s Hospital, Athens, Greece

**Keywords:** Head trauma, tremor, dystonia, diffuse axonal injuries

## Abstract

Despite the fact that movement disorders can be noted after severe traumatic brain injury (TBI), there are very few cases in the literature regarding children with a mild head injury. In addition, their pathophysiological mechanism, radiological features, and treatment options have not been well described yet. Hereby, we report a case of a 3-year-old girl who suffered a head injury after a two-meter fall, resulting in generalized body tremor and dystonia.

## Introduction

Post-traumatic movement disorders have been reported after severe craniocerebral injuries, mainly after traffic accidents, with dystonia, tremor and Parkinsonism being the most commonly reported [1]. Little is known regarding the prevalence or the prognosis, especially in the pediatric population, where a great variety of symptoms can occur, even after a long period of time. A high clinical suspicion is of utmost importance in order not to misdiagnose such disorders, particularly in children with a history of mild or even minor head injury.

## Patient and observation

**Figure 1:**
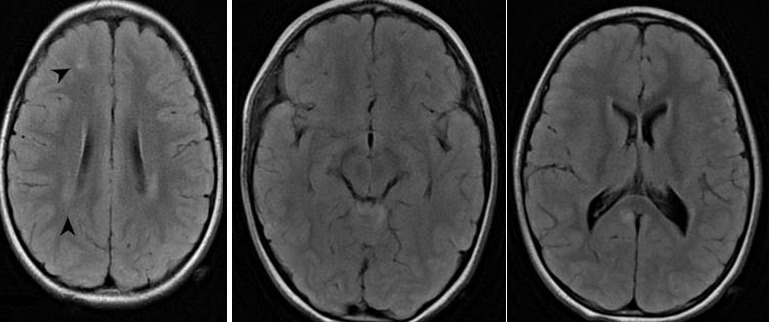
Sagittal T2 FLAIR High signal changes indicating diffuse axonal injuries in corpus callosum, cerebellar, and basal ganglia of the right hemisphere

**Figure 2:**
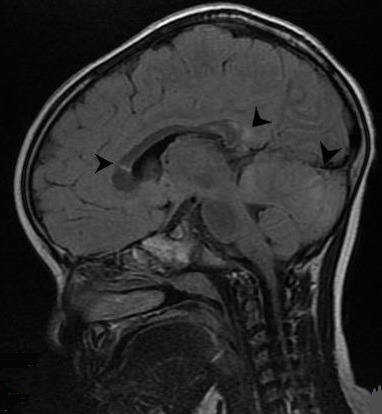
Sagittal T2 FLAIR High signal changes indicating diffuse axonal injuries in corpus callosum, cerebellar, and basal ganglia of the right hemisphere

A 3-year-old girl was admitted to our emergency department, with a history of falling from a 2-meter high bed while playing, crushing the right side of her head on a marble floor, approximately one hour before admission. During the initial examination, the child was crying, moving her arms and legs, but did not respond to verbal stimuli. In addition, two episodes of vomiting have been reported. The GCS on admission was 12(E:2, M: 5, V:5 ). Gradually, in the next two hours, the patient's breathing became irregular and response to stimuli worsened (GCS:7 E:1, M:3, V:3); hence the child was intubated and underwent an emergency brain and cervical spine CT scan, which revealed no pathological findings. She was then admitted to the intensive care unit. A catheter for intracranial pressure monitoring was placed. The initial ICP was 14mmHg and remained between the normal values the following day. When the patient was awakened, next day, tremor of torso, arms and legs was present. Neurological examination revealed hyperactive deep tendon reflexes, neck stiffness and positive Babinski sign on right foot. Right pupil was slightly dilated. The patient started treatment with levetiracetam 40mg/kg/d, Clobazam 1,5mg/kg/d and dexmedetomidine 0,2g/kg/h. A brain MRI revealed high signal changes on T2W and flair sequences, at the junction of gray and white matter, in both hemispheres, but mainly in the right frontal lobe, the right cerebellar hemisphere, the vermis and the splenium of corpus callosum (Figure 1, Figure 2). The lesions have a round shape, which is commonly described as “axonal bulb” or “retraction ball” and indicates diffuse axonal injuries (DAI), due to shear-strain deformations of brain tissue, and also the subacute phase of the injury [2]. The lesions located in the corpus callosum and basal ganglia on the right side are suggested to play a significant role regarding the presence of both tremor and dystonia. In addition, the diffuse axonal injuries in the cerebellar hemispheres and vermis may have contributed to the appearance of tremor [3, 4]. No pathological findings were present on spine MRI. The patient remained in the intensive care unit for two more days and then returned to our neurosurgical department. She was discharged home three days later with significant improvement of tremor. In the next six months the child was winning off the drugs and in one year follow up she is free of symptoms with no drugs.

## Discussion

Diffuse axonal injuries have initially been found to be responsible for severe complications of brain injury, such as coma and death. Nowadays, it is believed that DAI can lead to a variety of health conditions [2], either early, or delayed, possibly due to ongoing inflammation in the white matter, with movement disorders being the most frequently recorded [3]. Thalamic, cerebellar lesions and lesions in the area of basal ganglia are known to play a critical role in the appearance of movement disorders [3]. Little are known regarding the prognosis of post-traumatic movement disorders. Medical treatment is the treatment of choice so far, with benzodiazepines, propranolol, anticholinergics and anticonvulsants being the most effective. Botulinum toxin injections offer an alternative choice, especially for patients with focal dystonia or persistant tremor. Deep brain stimulation (DBS) is used in more severe cases, caused mainly after severe traumatic injuries [1, 3, 5].

## Conclusion

There are only a few reports in the literature regarding dystonia and tremor after head injury and diffuse axonal injury, with most of them referring to cases of severe traumatic brain injury. However, white matter tracts are prone to injuries even after mild head trauma, leading to movement disorders that often do not sufficiently respond to medical treatment [2, 3].

## Competing interests

The authors declare no competing interests.

## Authors’ contributions

All the authors have read and agreed to the final manuscript.

## References

[1] Johnson SL, Hall DM (1992). Post-traumatic tremor in head injured children. Archives of disease in childhood.

[2] Johnson VE, Stewart W, Smith DH (2013). Axonal pathology in traumatic brain injury. Experimental neurology.

[3] Krauss JK (2015). Movement disorders secondary to craniocerebral trauma. Handbook of clinical neurology.

[4] Puschmann A, Wszolek ZK (2011). Diagnosis and treatment of common forms of tremor. Seminars in neurology.

[5] Biary N, Cleeves L, Findley L, Koller W (1989). Post-traumatic tremor. Neurology.

